# Bounding the per-protocol effect in randomized trials: an application to colorectal cancer screening

**DOI:** 10.1186/s13063-015-1056-8

**Published:** 2015-11-30

**Authors:** Sonja A. Swanson, Øyvind Holme, Magnus Løberg, Mette Kalager, Michael Bretthauer, Geir Hoff, Eline Aas, Miguel A. Hernán

**Affiliations:** Department of Epidemiology, Erasmus Medical Center, PO Box 2040, 3000 CA Rotterdam, The Netherlands; Department of Epidemiology, Harvard TH Chan School of Public Health, Boston, MA USA; Institute of Health and Society, University of Oslo, Oslo, Norway; Sørlandet Hospital Kristiansand, Kristiansand, Norway; Telemark Hospital, Skien, Norway; Oslo University Hospital, Oslo, Norway; Cancer Registry of Norway, Oslo, Norway; Department of Biostatistics, Harvard TH Chan School of Public Health, Boston, MA USA; Harvard-MIT Division of Health Sciences and Technology, Boston, MA USA

**Keywords:** Instrumental variable, Partial identification, Per-protocol effect, Colorectal cancer, Screening

## Abstract

**Background:**

The per-protocol effect is the effect that would have been observed in a randomized trial had everybody followed the protocol. Though obtaining a valid point estimate for the per-protocol effect requires assumptions that are unverifiable and often implausible, lower and upper bounds for the per-protocol effect may be estimated under more plausible assumptions. Strategies for obtaining bounds, known as “partial identification” methods, are especially promising in randomized trials.

**Results:**

We estimated bounds for the per-protocol effect of colorectal cancer screening in the Norwegian Colorectal Cancer Prevention trial, a randomized trial of one-time sigmoidoscopy screening in 98,792 men and women aged 50–64 years. The screening was not available to the control arm, while approximately two thirds of individuals in the treatment arm attended the screening. Study outcomes included colorectal cancer incidence and mortality over 10 years of follow-up. Without any assumptions, the data alone provide little information about the size of the effect. Under the assumption that randomization had no effect on the outcome except through screening, a point estimate for the risk under no screening and bounds for the risk under screening are achievable. Thus, the 10-year risk difference for colorectal cancer was estimated to be at least −0.6 % but less than 37.0 %. Bounds for the risk difference for colorectal cancer mortality (–0.2 to 37.4 %) and all-cause mortality (–5.1 to 32.6 %) had similar widths. These bounds appear helpful in quantifying the maximum possible effectiveness, but cannot rule out harm. By making further assumptions about the effect in the subpopulation who would not attend screening regardless of their randomization arm, narrower bounds can be achieved.

**Conclusions:**

Bounding the per-protocol effect under several sets of assumptions illuminates our reliance on unverifiable assumptions, highlights the range of effect sizes we are most confident in, and can sometimes demonstrate whether to expect certain subpopulations to receive more benefit or harm than others.

**Trial registration:**

Clinicaltrials.gov identifier NCT00119912 (registered 6 July 2005)

**Electronic supplementary material:**

The online version of this article (doi:10.1186/s13063-015-1056-8) contains supplementary material, which is available to authorized users.

## Background

Most randomized trials report the intention-to-treat (ITT) effect as the primary, or only, measure of the comparative effect of the studied interventions. A focus on the ITT effect is attractive for several reasons [1, 2]. However, the ITT effect may not be the effect of interest for patients and clinicians when there is a high rate of non-compliance or when the rate of non-compliance in the trial differs from that expected outside the trial setting. In such circumstances, the per-protocol effect – the effect that would have been observed had all trial participants followed the trial protocol – may be of greater interest [1, 2]. Unfortunately, when patient characteristics associated with non-compliance are also related to patient outcomes, the naïve approach to estimating this effect in a “per-protocol analysis” restricted to those who follow the protocol in each arm of the trial will be biased. In these cases, identifying the per-protocol effect in a randomized trial requires strong assumptions (e.g., no unmeasured confounding) and methods that are commonly used in the analysis of non-randomized studies [2].

An alternative to estimating the per-protocol effect under these strong assumptions is to estimate lower and upper limits or “bounds” for the per-protocol effect under weaker, but perhaps more realistic, assumptions [3–8]. While effect bounding, known as “partial identification of the effect”, has been attempted in observational studies (particular in the social sciences), it is rarely implemented in randomized trials. This is surprising because partial identification methods can capitalize on assumptions that are expected to hold in many randomized trials.

Here we provide a guide to the use of partial identification methods in randomized trials with dichotomous outcomes and point interventions, i.e., interventions that are not sustained over time. As an example, we demonstrate the estimation of bounds for the per-protocol effect of colorectal cancer (CRC) screening on the 10-year risk of CRC incidence and death in the Norwegian Colorectal Cancer Prevention (NORCCAP) trial.

## Methods

### The NORCCAP trial

The design, procedures, and primary findings of the NORCCAP trial have been described elsewhere [9–11]. In brief, 98,792 residents of the City of Oslo and of Telemark County, Norway, who had no history of CRC and were aged 55–64 years in 1998 or 50–54 years in 2000 were randomly assigned to either treatment or control arms. Those selected for the treatment arm were invited to CRC screening, including either a once-only flexible sigmoidoscopy or a combination of once-only flexible sigmoidoscopy plus an immunochemical fecal occult blood testing. Individuals assigned to the control arm were not offered any intervention. All participants who attended the screening provided written informed consent, and the study was approved by the Ethics Committee of South-East Norway and the Norwegian Data Inspectorate. Primary study endpoints pre-specified in the study protocol were CRC incidence and mortality. Table 1 shows the 10-year risk of these outcomes [10] by age group, randomization arm, and screening intervention received. After standardization by age group, the ITT 10-year risk differences (95 % confidence interval) were −0.2 % (−0.4 %, −0.1 %) for CRC incidence, −0.1 % (−0.1 %, 0.0 %) for CRC mortality, and −0.2 % (−0.6 %, 0.2 %) for all-cause mortality.

**Table 1:** 10-year risk of colorectal cancer (CRC), CRC mortality, and all-cause mortality by randomization arm and treatment received

Randomization arm	Received treatment	*N*	CRC cases, *N* (%)	Cases of CRC mortality, *N* (%)	Cases of all-cause mortality, *N* (%)
Aged 50–54 years					
No screening^a^	No screening	37,131	297 (0.8)	78 (0.2)	2245 (6.0)
Screening	No screening	2811	18 (0.6)	7 (0.3)	260 (9.2)
	Screening	4109	19 (0.5)	5 (0.1)	141 (3.4)
Aged 55–64 years					
No screening^a^	No screening	41,089	593 (1.4)	180 (0.4)	4209 (10.2)
Screening	No screening	4806	74 (1.5)	34 (0.7)	779 (16.2)
	Screening	8846	97 (1.1)	17 (0.2)	592 (6.7)

^a^Screening was not available for individuals in the no-screening randomization arm

Several features of this trial are relevant for our purposes. First, CRC screening was not available in the trial communities for individuals not assigned to screening in the trial; thus, nobody in the control arm received it. Second, the treatment was a once-only screen (a point intervention); thus, compliance in the screening arm is all-or-nothing. Third, loss-to-follow-up was minimal; only 3 individuals were not followed until they experienced a study endpoint, emigration, or end of 10-year follow-up.

### Overview of the analytic approach

The following sections describe the estimation of bounds for the per-protocol effect of point interventions on dichotomous outcomes under increasingly stronger assumptions. We begin with no assumptions (i.e., the data alone), then assume the so-called “instrumental conditions” described below, and then combine additional assumptions with these instrumental conditions. Intuitively, the more assumptions we make, the narrower the bounds become, but of course this comes at a cost if our assumptions are ill-placed. Table 2 summarizes the assumptions.

**Table 2:** Description of analytic assumptions

Conditions^a^	Description of condition	Empirically verifiable in a randomized trial?	When the condition may be reasonable
Relevance (instrumental condition 1)^b^	Randomization indicator is associated with treatment	Yes	Expected to hold in randomized trials, and is empirically verifiable
Exclusion restriction (instrumental condition 2)^b^	Randomization indicator has no effect on the outcome except through treatment	No	Expected to hold in double-blinded placebo-controlled trials when double-blinding is successfully maintained and there is no placebo effect; may be approximately reasonable in other settings
Exchangeability (instrumental condition 3)^b^	The effect of the randomization indicator on the outcome is not confounded	No	Expected to hold by design if no loss to follow-up or other forms of selection bias occur
Known feasible compliance type distribution (e.g., no “defiers”)	Specify the proportion of patients that are “compliers,” “always-takers,” “never-takers,” or “defiers”	No	Compliance type distribution is identified in trials where non-adherence only occurs in one arm (e.g., when treatment is not available to the placebo arm); assuming zero or a minimal number of “defiers” may be reasonable in other settings
Limits on the unobserved compliance type counterfactual risks^c^	Specify imposed limits on what could happen to the “never-takers” had they been treated and the “always-takers” had they not been treated	No	Subject-matter dependent; imposed limits may be more justifiable for rare outcomes
Additive effect homogeneity	No additive effect modification by randomization arm among the treated and untreated	No	Subject-matter dependent and generally not expected to hold by design; may become more plausible in analyses conditional on measured patient characteristics
Multiplicative effect homogeneity	No multiplicative effect modification by randomization arm among the treated and untreated	No	Subject-matter dependent and generally not expected to hold by design; may become more plausible in analyses conditional on measured patient characteristics

^a^Bounds for the per-protocol effect presented in the current study rely on (1) no assumptions (data only); (2) the instrumental conditions; (3) the instrumental conditions, a feasible known distribution of compliance types, and imposed limits on unobserved compliance type counterfactual risks; (4) the instrumental conditions and additive effect homogeneity; and (5) the instrumental conditions and multiplicative effect homogeneity

^b^Relevance, exclusion restriction, and exchangeability are jointly referred to as the instrumental conditions

^c^In the NORCCAP trial, there are no “always-takers” by design and, therefore, we only discuss this assumption type in the context of the “never-takers”

To compute the bounds in the NORCCAP trial, we first estimated bounds within age groups (50–54 years; 55–64 years) and then standardized these bounds. We begin by estimating the bounds in the older age group only so readers can check our calculations using the expressions provided in the text and the summary data in Table 1.

## Results

### Bounding the per-protocol effect under no assumptions

The per-protocol risk difference can theoretically range from −100 % (the treatment universally prevents the outcome) to 100 % (the treatment universally causes the outcome). However, the study data – without any assumptions – can be used to exclude parts of the theoretical range of the per-protocol effect. To see this, consider that, for every person in the study, we observe one of their two potential or counterfactual outcomes: e.g., for those who were screened, we see their outcome under screening, but do not know what would have happened to them had they not been screened (see illustration in Additional file 1: Table S1). We can compute bounds for the per-protocol effect by imputing the most extreme scenarios for the unobserved counterfactual outcomes – e.g., for the upper bound, imagining that everybody who was screened would have experienced the outcome had they not been screened, and that everybody who was not screened would have not experienced the outcome had they been screened. Thus, the per-protocol risk difference must lie within the bounds (using probability notation):

$$ LB=\left( \Pr \left[Y=1\Big|X=1\right]-1\right) \Pr \left[X=1\right]- \Pr \left[Y=1\Big|X=0\right] \Pr \left[X=0\right] $$

$$ UB= \Pr \left[Y=1\Big|X=1\right] \Pr \left[X=1\right]+\left(1- \Pr \left[Y=1\Big|X=0\right]\right) \Pr \left[X=0\right], $$

where *X* is an indicator of treatment, *Y* is an indicator of a dichotomous outcome of interest, and LB and UB denote the lower and upper bounds, respectively. Expressions for the risk under each level of treatment, the risk ratio, and a formal definition of the effect of interest are provided in the Additional file 1.

The first block of Table 3 shows that these assumption-free bounds for the 10-year risk difference in the NORCCAP trial are quite wide: e.g., from −10.0 to 90.0 % for CRC incidence. In fact, these bounds will always cover the null and necessarily have a width of 100 % for dichotomous outcomes. In order to obtain narrower bounds for the per-protocol effect we need to combine the data with assumptions.

**Table 3:** Lower and upper bounds for 10-year counterfactual risks and per-protocol effects among individuals 55–64 years old (units = cases/100 persons for risks and risk differences)

	CRC incidence		CRC mortality		All-cause mortality	
	Lower bound	Upper bound	Lower bound	Upper bound	Lower bound	Upper bound
No Assumptions						
Risk under no screening	1.2	17.4	0.4	16.6	9.1	25.3
Risk under screening	0.2	84.0	0.03	83.9	1.1	84.9
Risk difference	−17.2	82.8	−16.5	83.5	−24.2	75.8
Risk ratio	0.01	68.95	0.00	214.54	0.04	9.32
Instrumental conditions						
Overall						
Risk under no screening^a^	1.4		0.4		10.2	
Risk under screening	0.7	35.9	0.1	35.3	4.3	39.5
Risk difference	−0.7	34.5	−0.3	34.9	−5.9	29.3
Risk ratio	0.49	24.88	0.28	80.64	0.42	3.86
Among the “never-takers” (35 %)^b^						
Risk under no screening^a^	1.5		0.7		16.2	
Risk under screening	0.0	100.0	0.0	100.0	0.0	100.0
Risk difference	−1.5	98.5	−0.7	99.3	−16.2	83.8
Risk ratio	0.00	64.95	0.00	141.35	0.00	6.17
Among the “compliers” (65 %)^a,b^						
Risk under no screening	1.4		0.3		7.0	
Risk under screening	1.1		0.2		6.7	
Risk difference	−0.3		−0.1		−0.3	
Risk ratio	0.79		0.66		0.96	
Instrumental conditions and additive effect homogeneity^a^						
Risk under no screening	1.4		0.4		10.2	
Risk under screening	1.1		0.3		9.9	
Risk difference	−0.3		−0.1		−0.3	
Risk ratio	0.80		0.77		0.97	
Instrumental conditions and multiplicative effect homogeneity^a^						
Risk under no screening	1.4		0.4		10.2	
Risk under screening	1.1		0.3		9.8	
Risk difference	−0.3		−0.1		−0.5	
Risk ratio	0.79		0.66		0.96	

^a^Point identification is achieved under these conditions in the NORCCAP trial

^b^In this particular study the distribution of compliance types is known given instrumental conditions. In other study designs, identifying the counterfactual risks and treatment effects within compliance types requires an additional assumption of an assumed feasible distribution of compliance types

### Bounding the per-protocol effect under the instrumental conditions

The three instrumental conditions are often described as follows: (1) the randomization indicator (which we will denote *Z*) is associated with receiving treatment *X*; (2) the randomization indicator *Z* causes the outcome *Y* only through treatment *X*; and (3) the randomization indicator *Z* and the outcome *Y* share no causes [3]. The first condition, sometimes referred to as the “relevance” condition, can be checked in the data: e.g., in the 55–64-year age group of the NORCCAP trial the risk difference:

$$ \Pr \left[X=1\Big|Z=1\right]- \Pr \left[X=1\Big|Z=0\right]=0.65-0=0.65. $$

Here, the F-statistic = 75626 and *p* value < 0.0001.

The third “exchangeability” condition is expected by design in randomized trials. The second condition, known as the exclusion restriction, is expected to hold in double-blinded placebo-controlled randomized trials in which double-blinding is successfully maintained and there is no placebo effect, but may not hold in other trials, like ours. For example, the exclusion restriction would be violated if the invitation letter alone prompted new awareness of CRC risk and risk factors, and subjects in the screening arm adopted preventive measures that they would not have adopted had they been in the control arm. Although we cannot prove conditions (2) and (3) hold in a given study, it is sometimes possible to find empirical evidence refuting, i.e., falsifying, them [3].

Under the instrumental conditions, we can compute the following bounds for the per-protocol risk difference:

$$ LB= \max \left(\begin{array}{c}\hfill \Pr \left[Y=1,X=1\Big|Z=1\right]+ \Pr \left[Y=0,X=0\Big|Z=0\right]-1,\hfill \\ {}\hfill \Pr \left[Y=1,X=1\Big|Z=0\right]+ \Pr \left[Y=0,X=0\Big|Z=1\right]-1,\hfill \\ {}\hfill \Pr \left[Y=1,X=1\Big|Z=0\right]- \Pr \left[Y=1,X=1\Big|Z=1\right]- \Pr \left[Y=1,X=0\Big|Z=1\right]- \Pr \left[Y=0,X=1\Big|Z=0\right]- \Pr \left[Y=1,X=0\Big|Z=0\right],\hfill \\ {}\hfill \Pr \left[Y=1,X=1\Big|Z=1\right]- \Pr \left[Y=1,X=1\Big|Z=0\right]- \Pr \left[Y=1,X=0\Big|Z=0\right]- \Pr \left[Y=0,X=1\Big|Z=1\right]- \Pr \left[Y=1,X=0\Big|Z=1\right],\hfill \\ {}\hfill - \Pr \left[Y=0,X=1\Big|Z=1\right]- \Pr \left[Y=1,X=0\Big|Z=1\right],\hfill \\ {}\hfill \kern0.5em - \Pr \left[Y=0,X=1\Big|Z=0\right]- \Pr \left[Y=1,X=0\Big|Z=0\right],\hfill \\ {}\hfill \Pr \left[Y=0,X=0\Big|Z=1\right]- \Pr \left[Y=0,X=1\Big|Z=1\right]- \Pr \left[Y=1,X=0\Big|Z=1\right]- \Pr \left[Y=0,X=1\Big|Z=0\right]- \Pr \left[Y=0,X=0\Big|Z=0\right],\hfill \\ {}\hfill \Pr \left[Y=0,X=0\Big|Z=0\right]- \Pr \left[Y=0,X=1\Big|Z=0\right]- \Pr \left[Y=1,X=0\Big|Z=0\right]- \Pr \left[Y=0,X=1\Big|Z=1\right]- \Pr \left[Y=0,X=0\Big|Z=1\right]\hfill \end{array}\right) $$

$$ UB= \min \left(\begin{array}{c}\hfill 1- \Pr \left[Y=0,X=1\Big|Z=1\right]+ \Pr \left[Y=1,X=0\Big|Z=0\right],\hfill \\ {}\hfill 1- \Pr \left[Y=0,X=1\Big|Z=0\right]+ \Pr \left[Y=1,X=0\Big|Z=1\right],\hfill \\ {}\hfill - \Pr \left[Y=0,X=1\Big|Z=0\right]+ \Pr \left[Y=0,X=1\Big|Z=1\right]+ \Pr \left[Y=0,X=0\Big|Z=1\right]+ \Pr \left[Y=1,X=1\Big|Z=0\right]+ \Pr \left[Y=0,X=0\Big|Z=0\right],\hfill \\ {}\hfill - \Pr \left[Y=0,X=1\Big|Z=1\right]+ \Pr \left[Y=0,X=1\Big|Z=0\right]+ \Pr \left[Y=0,X=0\Big|Z=0\right]+ \Pr \left[Y=1,X=1\Big|Z=1\right]+ \Pr \left[Y=0,X=0\Big|Z=1\right],\hfill \\ {}\hfill \Pr \left[Y=1,X=1\Big|Z=1\right]+ \Pr \left[Y=0,X=0\Big|Z=1\right],\hfill \\ {}\hfill \kern0.5em  \Pr \left[Y=1,X=1\Big|Z=0\right]+ \Pr \left[Y=0,X=0\Big|Z=0\right],\hfill \\ {}\hfill - \Pr \left[Y=1,X=0\Big|Z=1\right]+ \Pr \left[Y=1,X=1\Big|Z=1\right]+ \Pr \left[Y=0,X=0\Big|Z=1\right]+ \Pr \left[Y=1,X=1\Big|Z=0\right]+ \Pr \left[Y=1,X=0\Big|Z=0\right],\hfill \\ {}\hfill - \Pr \left[Y=1,X=0\Big|Z=0\right]+ \Pr \left[Y=1,X=1\Big|Z=0\right]+ \Pr \left[Y=0,X=0\Big|Z=0\right]+ \Pr \left[Y=1,X=1\Big|Z=1\right]+ \Pr \left[Y=1,X=0\Big|Z=1\right]\hfill \end{array}\right) $$

Related bounds have also been proposed under different interpretations of condition (3) [6, 8], but the bounds presented here make use of the strongest version of this condition expected to hold in randomized trials. See Richardson and Robins [12] for further discussion, including some intuition for these complicated expressions. See Additional file 1 for bounds for the (counterfactual) absolute risks under each treatment.

The second block of Table 3 shows that the bounds for the 10-year risk difference under the instrumental conditions are quite wide in the NORCCAP trial. For example, for CRC risk, the effect may fall anywhere between –0.7 % and 34.5 %. Interestingly, we can obtain a point estimate for the risk under no screening (for CRC risk: 1.4 %) but, because there is non-compliance in the screening arm, only bounds for the risk under screening (for CRC risk: 0.7 to 35.9 %). The wide bounds for the risk under screening drive the wide bounds for the risk difference.

### Bounding the per-protocol effect within compliance types

Under the instrumental conditions, we can describe patients in the study population as belonging to one of four mutually-exclusive “compliance types” or “principal strata” [13–15]:

1. “Always-takers,” those who would have always been treated regardless of randomization
2. “Never-takers,” those who would have always opted out of treatment regardless of randomization
3. “Compliers,” those who would have been treated had they been randomized to receive treatment, and would not have been treated had they been randomized to the control arm
4. “Defiers,” those who would not have been treated had they been randomized to receive treatment, but would have been treated had they been randomized to the control arm

For many trials, it may be reasonable to assume there are zero (or at least a small number of) “defiers.” If we assume there are no “defiers” then we can identify the proportion of our study population who are in each of the other compliance types.

In the NORCCAP trial, there are no “always-takers” and no “defiers” because the screening was not available to those who were randomized to the control arm. Therefore, under exchangeability of the randomization arms, 35 % of trial participants are “never-takers” (estimated by Pr [*X* = 0|*Z* = 1]) and the other 65 % are “compliers”. For each person in the treatment arm we know whether she is a “complier” (if she did undergo screening) or a “never-taker” (if she did not). In studies that have non-compliance in both randomization arms, we will not know with certainty any given subject’s compliance type.

Richardson and Robins [5] described bounds for the counterfactual risks and treatment effects within compliance types. In the special case when we (i) know there are only “compliers” and “never-takers” and (ii) have no empirical evidence against the instrumental conditions, then the effect within the “never-takers” is bounded as follows:

$$ UB=1- \Pr \left[Y=1\Big|X=0,Z=1\right] $$

$$ LB=- \Pr \left[Y=1\Big|X=0,Z=1\right]. $$

Meanwhile, the effect in the “compliers” is point-identified:

$$ LB=UB=\frac{ \Pr \left[Y=1\Big|Z=1\right]- \Pr \left[Y=1\Big|Z=0\right]}{ \Pr \left[X=1\Big|Z=1\right]- \Pr \left[X=1\Big|Z=0\right]}\ . $$

Beyond the special case where (i) is expected by design, more general expressions have been described for an assumed distribution of compliance types [5]. Note that any assumed distribution needs to be feasible. For example, in studies like the NORCCAP trial with non-compliance in only one treatment arm, the only feasible proportion of “defiers” is zero. In trials with non-compliance in both treatment arms, the data may be consistent with a range in the proportion of “defiers,” and investigators may consider computing bounds for the effects within compliance types under an assumed proportion of “defiers” within that range.

The second block of Table 3 further presents bounds for the counterfactual risks and per-protocol effect within the “compliers” and the “never-takers.” (In the NORCCAP trial, there is no evidence against the instrumental conditions, so we can use the expressions described above.) For the “never-takers”, we can obtain a point estimate for the risks under no screening, but we have (by definition) no information on what would have happened to them had we forced them to follow the protocol in the screening arm and, therefore, been screened. Therefore, we can only achieve wide bounds for the per-protocol effect in the “never-takers” because we have limited information in the data on what would have happened to this subgroup had they followed the protocol. For the “compliers”, we can obtain a point estimate for the risks under screening and no screening, and, therefore, we can also obtain a point estimate for the per-protocol effect in the “compliers” [10], often referred to as a “local” average treatment effect [15]. In the NORCCAP trial, the “compliers” are known to be those who actually received the screening, and thus the effect in the “compliers” is the effect in the screened.

**Fig. 1:**
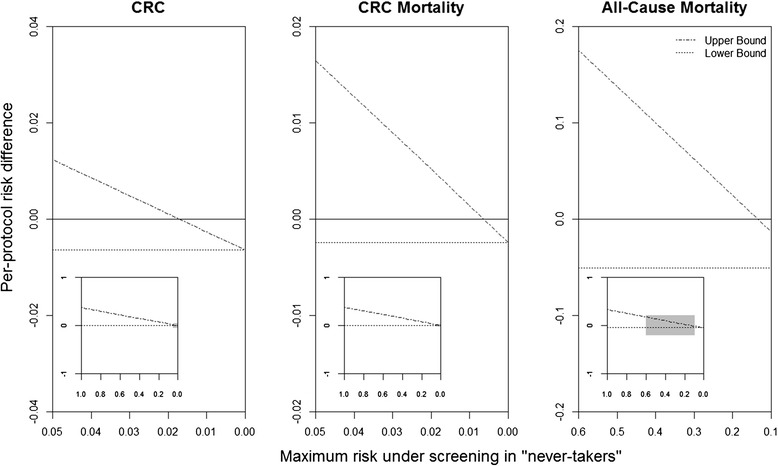
Bounds for the per-protocol 10-year risk difference when restricting the maximum value of the risk under screening in the “never-takers”, aged 55–64 years. Gray area in nested plots indicates the area of detail presented in the outer plots

The bounds in the NORCCAP trial using the instrumental conditions alone, described above, are a weighted average of the bounds in the “never-takers” and the point estimate in the “compliers”. In order to obtain narrower bounds for the per-protocol effect in the study population, we may combine the instrumental conditions with restrictions on the upper bound of the risk under screening in the “never-takers” (i.e., a counterfactual risk in 35 % of the study population for which we have no empirical information; Fig. 1) [5]. For example, we might assume that the risk under screening in the “never-takers” is actually not greater than their risk under no screening. Under this assumption, the resulting bounds do not include the null value: (−0.7 %, −0.2 %) for CRC risk, (−0.3 %, −0.1 %) for CRC mortality, and (−5.9 %, −0.2 %) for all-cause mortality. Though this assumption is plausible for CRC risk and mortality, it may not be for all-cause mortality, which could be more susceptible to unintended consequences of screening. A less stringent restriction for all-cause mortality is that at most, say, 50 % of the “never-takers” would have died had they been screened. As seen in Fig. 1, this restriction (*x*-axis = 0.5) would imply bounds that include the possibility of a null or positive risk difference. A sensitivity analysis can be conducted under different hypothesized risks under screening in the “never-takers”, or the full range of possibilities could be presented as we do in the insets in Fig. 1. For trials with non-compliance in both treatment arms, similar sensitivity analyses could be conducted under hypothesized risks under no treatment in the “always-takers”.

### Point identification of the per-protocol effect under the instrumental conditions plus homogeneity

The bounds can also be narrowed by combining the instrumental conditions with assumptions that restrict effect heterogeneity. In fact, when the instrumental conditions are combined with sufficiently strong homogeneity assumptions, a point estimate of the per-protocol effect can be obtained [6, 7, 16]. Assuming (i) no *additive* effect modification by the instrument among the treated and the untreated leads to what is often referred to as the standard instrumental variable (IV) estimator:

$$ \frac{ \Pr \left[Y=1\Big|Z=1\right]- \Pr \left[Y=1\Big|Z=0\right]}{ \Pr \left[X=1\Big|Z=1\right]- \Pr \left[X=1\Big|Z=0\right]}. $$

Assuming (ii) no *multiplicative* effect modification by the instrument among the treated and the untreated leads to a different estimator:

$$ \Pr \left[Y=1\Big|X=0\right] \Pr \left[X=0\right]\left( \exp \left(\psi \right)-1\right)+ \Pr \left[Y=1\Big|X=1\right] \Pr \left[X=1\right]\left(1- \exp \left(-\psi \right)\right), $$

where

$$ \exp \left(-\psi \right)=1-\frac{ \Pr \left[Y=1\Big|Z=1\right]- \Pr \left[Y=1\Big|Z=0\right]}{ \Pr \left[Y=1\Big|X=1,Z=1\right] \Pr \left[X=1\Big|Z=1\right]- \Pr \left[Y=1\Big|X=1,Z=0\right] \Pr \left[X=1\Big|Z=0\right]}. $$

In the NORCCAP trial, because we have only “compliers” and “never-takers”, these assumptions could be restated as equal effects in the “compliers” and “never-takers” on the (i) additive or (ii) multiplicative scale. Effect homogeneity assumptions may be implausible in many trials, particularly if there are interactions between patients’ treatment assignment and characteristics in informing treatment choice [16].

The third and fourth blocks of Table 3 shows the point estimates of the per-protocol risk difference under effect homogeneity on the additive and multiplicative scale, respectively. These assumptions will not hold in our study if, for example, family history of cancers modifies the effect of screening and patients in the screening arm with no family history of cancers may be more likely to forgo screening. The possibility for such modification is also apparent when examining the baseline risks across compliance types: the risk under no screening is lower in the “compliers” than the “never-takers” which might indicate the magnitude of the effects in the “compliers” and “never-takers” could be very different. If family history and other relevant patient characteristics were measured, it would be possible to relax the homogeneity assumptions (and instrumental conditions) to hold within levels of covariates and then present a point estimate of the per-protocol effect within levels of the covariates [6, 7, 16].

### Bounds and point identification results for the per-protocol effect, aged 50–64 years

**Fig. 2:**
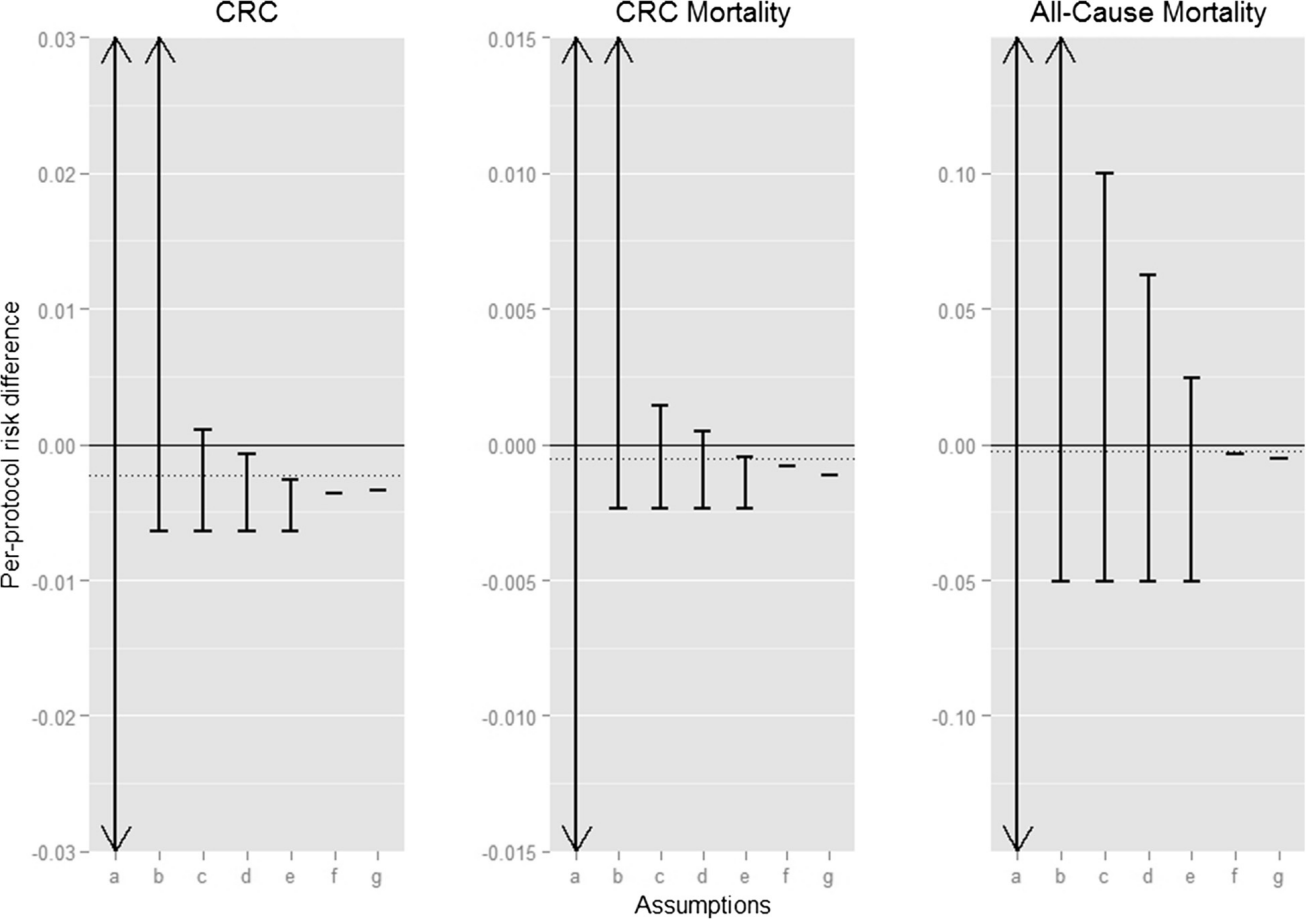
Age-standardized bounds (for ages 50–64) for the per-protocol 10-year risk difference under various sets of assumptions. Sets of assumptions include: **a** No assumptions. **b** The instrumental conditions (relevance, exclusion restriction, and exchangeability). **c** The instrumental conditions plus an assumed maximum risk under screening in the “never-takers” of 2 %, 1 %, and 40 % for the CRC incidence, CRC mortality, and all-cause mortality, respectively. **d** The instrumental conditions plus an assumed maximum risk under screening in the “never-takers” of 1.5 %, 0.75 %, and 30 % for the CRC incidence, CRC mortality, and all-cause mortality, respectively. **e** The instrumental conditions plus an assumed maximum risk under screening in the “never-takers” of 1 %, 0.5 %, and 20 % for the CRC incidence, CRC mortality, and all-cause mortality, respectively. **f** The instrumental conditions plus additive effect homogeneity. **g** The instrumental conditions plus multiplicative effect homogeneity. The dotted line indicates the intention-to-treat effect

**Fig. 3:**
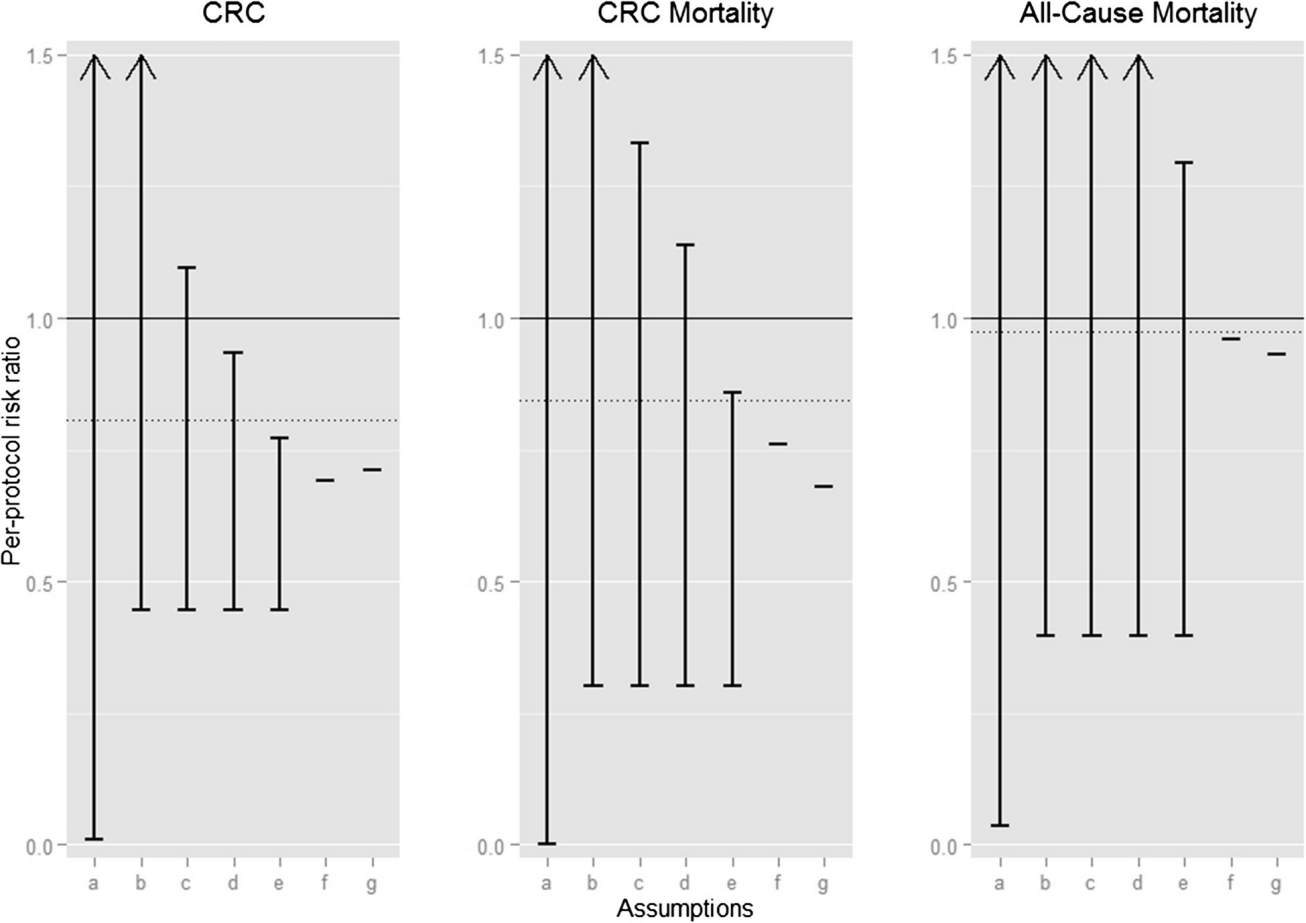
Age-standardized bounds (for ages 50–64) for the per-protocol 10-year risk ratio under various sets of assumptions. Sets of assumptions include: **a** No assumptions. **b** The instrumental conditions (relevance, exclusion restriction, and exchangeability). **c** The instrumental conditions plus an assumed maximum risk under screening in the “never-takers” of 2 %, 1 %, and 40 % for the CRC incidence, CRC mortality, and all-cause mortality, respectively. **d** The instrumental conditions plus an assumed maximum risk under screening in the “never-takers” of 1.5 %, 0.75 %, and 30 % for the CRC incidence, CRC mortality, and all-cause mortality, respectively. **e** The instrumental conditions plus an assumed maximum risk under screening in the “never-takers” of 1 %, 0.5 %, and 20 % for the CRC incidence, CRC mortality, and all-cause mortality, respectively. **f** The instrumental conditions plus additive effect homogeneity. **g** The instrumental conditions plus multiplicative effect homogeneity. The dotted line indicates the intention-to-treat effect

Thus far, we have considered bounding the counterfactual risks and the per-protocol effect among the 55–64-year age group. We repeated these computations for subjects aged 50–54 years (Additional file 1: Table S2). We then standardized by age group to obtain the estimates presented in Additional file 1: Table S3; sex-stratified results are presented in Additional file 1: Table S4. The final age-standardized bounds for the per-protocol risk difference and risk ratio estimated under each set of assumptions are shown in Figs. 2 and 3. As demonstrated above with estimating the effect among the 55–64-year age group, the age-standardized bounds for the risk difference under the instrumental conditions are relatively wide (e.g., the risk difference for CRC incidence is between −0.6 % and 37.0 %), while narrower bounds or even point identification can be achieved by making additional assumptions about the possible magnitude and direction of the effect in the “never-takers”.

## Discussion

We have demonstrated how combining data with various sets of assumptions helps to bound the per-protocol effect of point interventions (i.e., interventions that are not sustained over time) in randomized trials with dichotomous outcomes. In our application to a trial of CRC screening, we showed how bounds for both the per-protocol risk difference and risk ratio are achievable. Our application illustrates three key benefits of an approach based on partial identification with progressively stronger assumptions.

First, this approach illuminates our reliance on unverifiable assumptions. In our trial, the wide bounds under no assumptions make clear that we cannot learn much at all about the effectiveness of screening without bringing in prior knowledge about the study design or our subject matter.

Second, this approach provides the range of effect sizes we are most confident in under fairly reasonable assumptions. In our trial we could estimate relatively informative lower bounds that quantify the maximum benefit of screening. For example, had everybody been screened, at most we would expect CRC risk to decrease by 0.6 percentage points. This number provides a limit for how much our ITT effect estimate (−0.2 %) might underestimate the effectiveness under perfect adherence, and a boundary that could be helpful in evaluating the cost-effectiveness of screening or informing clinical or policy decisions. We know less about the upper bound (minimum effectiveness or even possible harm) of the screening program without making more debatable assumptions, but the type of analyses presented in Fig. 1 provides a template for discussing what level of assumptions may be reasonable and how much differing opinions may lead to differing conclusions.

Third, this approach can demonstrate our confidence, or lack thereof, in the effect sizes for certain subpopulations [13–15]. In our trial, the estimates support the benefit of CRC screening for nearly two thirds of the study population (the “compliers”), and in this case we can describe which individuals are included in this group. In randomized trials with non-compliance in both arms, we can only obtain a point estimate for the effect in the “compliers” if we assume there are no “defiers”. However, we would not know who the “compliers” are and membership in this group may vary across studies. Because of this, the common practice of presenting this subgroup effect alone is of questionable interest for clinical or policy decision-making [17] as there is no obvious way of applying the results of the study to that particular subgroup. When presented alongside bounds for the effect in the full study population, however, investigators may sometimes be able to discern whether certain subpopulations are likely to receive more benefit or harm than others. In trials with one-sided non-compliance, like the NORCCAP trial, such practice is sometimes actionable because we can describe the subpopulation of “compliers” based on measured pre-randomization characteristics.

Investigators considering employing these methods in randomized trials with point interventions and dichotomous outcomes should consider how features of their particular study design may affect which sets of assumptions we describe in Table 2 are reasonable. The instrumental conditions are expected to hold in placebo-controlled, double-blinded randomized trials of point interventions where there is no loss to follow-up, no placebo effect, and double-blinding is successfully maintained, but the instrumental conditions are suspect in head-to-head randomized trials and whenever double-blinding is not successfully maintained or there is a possible placebo effect. The homogeneity conditions, on the other hand, are not expected to hold based on any study design feature and thus should be weighed judiciously when applied to the analysis of any randomized trial. A similar caveat applies to conditions about the distribution of or effects within compliance types when there is non-compliance in both treatment arms [5].

Our discussion of bounding the per-protocol effect focused on dichotomous outcomes and point interventions. Similar bounds under the instrumental conditions can be identified for continuous outcomes if one assumes the outcomes are finitely bounded [8], and the point-identification expressions under effect homogeneity conditions can also be restated to apply to continuous outcomes [6, 7, 16]. Because we can choose to estimate cumulative risk up through any point in time in follow-up, we could also extend these bounds to bounding the survival curve for time-to-event outcomes [18]. Partial identification strategies can also be applied to trials with substantial attrition by further incorporating methods to account for selection bias, e.g., inverse probability weighting [19]. In trials that involve an intervention sustained over time, accounting for non-adherence can be more complicated as participants may discontinue the intervention at different times during follow-up and time-varying patient characteristics may inform and be affected by these decisions. More research is needed on how to generalize partial identification strategies to such settings, although the point-identification results can be expanded upon using structural nested models under related homogeneity and instrumental conditions [7, 20]. Finally, our example and discussion has focused on identification, but there is a growing body of literature on how to incorporate random variability [21]. Specifically, there has been recent development in methods for estimating confidence intervals around the bounds [22–26] as well as estimating confidence intervals for the partially identified treatment effect itself [27, 28]. Incorporating random variability into the presentation of partial identification results in randomized trials is critical; however, more research is needed as there is currently no consensus in the statistical literature on – or readily available software for – the optimal approach.

The per-protocol effect is often of greater interest than, or complementary with, the ITT effect [1, 2]. In trials like the NORCCAP trial with essentially no loss to follow-up, we can easily compute an unbiased estimate for the ITT effect. However, the ITT effect quantifies the effect of assignment to treatment. From a patient’s perspective, deciding whether or not to take treatment requires knowledge about the effect of the treatment when received as intended rather than the effect of merely being assigned to treatment [1, 2]. Further, the ITT effect is study-specific because it depends on the magnitude and type of observed adherence to the intervention among study participants. That the per-protocol effect is independent of the observed adherence makes it interesting from a societal perspective too. For example, were the screening made available in the future to the Norwegian population, the actual adherence to the intervention could be different from that observed in the trial (not the least because the trial itself contributed to establish the efficacy of screening). As a result, the ITT effect from the trial would be outdated as a tool for decision-making, e.g., for cost-effectiveness analyses. On the other hand, unbiased estimates for the per-protocol effect, while potentially more relevant for decision making, are not achievable from the data alone: investigators need to combine the data with assumptions based on the study design and subject matter expertise. Historically, this has deterred many investigators from estimating the per-protocol effect as expert knowledge is, by definition, provisional and fallible.

## Conclusion

As we have demonstrated using data from the NORCAPP trial, bounding the per-protocol effect under several sets of assumptions provides investigators with a middle ground between presenting a single value for the per-protocol effect based on sometimes heroic assumptions versus avoiding estimating the per-protocol effect altogether. This middle ground shifts the scientific debate to what assumptions are most plausible and, therefore, to what range of effect sizes we are most confident in.

## Additional file

Additional file 1:
**Norwegian Colorectal Cancer Prevention trial instrumental variable (NORCCAP IV) bounds trials submission supplement.** Supplemental tables and an appendix describing the derivations for bounds not presented in the main text. (PDF 132 kb)

## Abbreviations

CRC: colorectal cancer
ITT: intention-to-treat
IV: instrumental variable
NORCCAP trial: Norwegian Colorectal Cancer Prevention trial
