# Supplementary material for: Bounding the per-protocol effect in randomized trials: an application to colorectal cancer screening
Source: Trials. 2015 Nov 30;16:541. doi: 10.1186/s13063-015-1056-8 (PMC4666083; doi:10.1186/s13063-015-1056-8)
Supplement: Additional file 1: — Norwegian Colorectal Cancer Prevention trial instrumental variable (NORCCAP IV) bounds trials submission supplement. Supplemental tables and an appendix describing the derivations for bounds not presented in the main text. (PDF 132 kb) [file 13063_2015_1056_MOESM1_ESM.pdf]

Supplemental Table 1. Illustration of dataset and observed counterfactual outcomes.

Subject	Randomization arm	Treatment	Outcome	Outcome under treatment [a]	Outcome under no treatment [a]
1	0	0	0	?	0
2	0	0	0	?	0
3	0	0	0	?	0
4	0	0	0	?	0
5	0	0	1	?	1
6	1	0	0	?	0
7	1	0	1	?	1
8	1	1	0	0	?
9	1	1	0	0	?
10	1	1	1	1	?

a. For individuals who received no treatment, we observe what would have happened to them under no treatment, but we do not know what their outcome would have been had they, counter to fact, been treated (indicated by the "?" in the table). For individuals who received treatment, we observe what would have happened to them under treatment, but we do not know what their outcome would have been had they, counter to fact, not been treated (similarly indicated by the "?" in the table). Without any further assumptions, the data cannot address what would have happened had everybody been treated versus had nobody been treated because we at most observe only one of these two counterfactual outcomes for each person.

Supplemental Table 2. Lower and upper bounds for 10-year counterfactual risks and per-protocol effects among individuals 50-54 years old (units= cases/100 persons for risks and risk differences)

	CRC Incidence		CRC Mortality		All-Cause Mortality	
	**Lower Bound**	**Upper Bound**	**Lower Bound**	**Upper Bound**	**Lower Bound**	**Upper Bound**
**No Assumptions**						
Risk under no screening	0.7	10.0	0.2	9.5	5.7	15.0
Risk under screening	0.04	90.7	0.01	90.7	0.3	91.0
Risk difference	-10.0	90.0	-9.5	90.5	-14.7	85.3
Risk ratio	0.00	126.86	0.00	469.97	0.02	16.00
**Instrumental Conditions**						
Overall						
Risk under no screening[a]	0.8		0.2		6.0	
Risk under screening	0.3	40.9	0.1	40.7	2.0	42.7
Risk difference	-0.5	40.1	-0.1	40.5	-4.0	36.6
Risk ratio	0.34	51.13	0.34	193.72	0.34	7.06
Among the "Never-Takers" (35%)[b]						
Risk under no screening[a]	0.6		0.2		9.2	
Risk under screening	0.0	100.0	0.0	100.0	0.0	100.0
Risk difference	-0.6	99.4	-0.2	99.8	-9.2	90.8
Risk ratio	0.00	156.17	0.00	401.57	0.00	10.81
Among the "Compliers" (65%)[a,b]						
Risk under no screening	0.9		0.2		3.9	
Risk under screening	0.5		0.1		3.4	
Risk difference	-0.4		-0.1		-0.4	
Risk ratio	0.51		0.66		0.89	
**Instrumental Conditions and Additive Effect Homogeneity**[a]						
Risk under no screening	0.8		0.2		6.0	
Risk under screening	0.4		0.1		5.6	
Risk difference	-0.4		-0.1		-0.4	
Risk ratio	0.44		0.70		0.93	
**Instrumental Conditions and Multiplicative Effect Homogeneity**[a]						
Risk under no screening	0.8		0.2		6.0	
Risk under screening	0.4		0.1		5.4	
Risk difference	-0.4		-0.1		-0.7	
Risk ratio	0.51		0.66		0.89	

a. Point identification is achieved under these conditions in the NORCCAP trial.

b. In this particular study the distribution of compliance types is known given instrumental conditions. In other study designs, identifying the counterfactual risks and treatment effects within compliance types requires an additional assumption of an assumed feasible distribution of compliance types.

Supplemental Table 3. Age-standardized lower and upper bounds for 10-year counterfactual risks and per-protocol effects (units= cases/100 persons for risks and risk differences)

	**CRC Incidence**		**CRC Mortality**		**All-Cause Mortality**	
	**Lower Bound**	**Upper Bound**	**Lower Bound**	**Upper Bound**	**Lower Bound**	**Upper Bound**
**No Assumptions**						
Risk under no screening	1.0	14.1	0.3	13.4	7.6	20.7
Risk under screening	0.1	87.0	0.02	86.9	0.7	87.6
Risk difference	-14.0	86.0	-13.4	86.6	-20.0	80.0
Risk ratio	0.00	87.88	0.00	287.15	0.04	11.55
**Instrumental Conditions**						
Overall						
Risk under no screening[a]	1.2		0.3		8.4	
Risk under screening	0.5	38.1	0.1	37.7	3.3	40.9
Risk difference	-0.6	37.0	-0.2	37.4	-5.1	32.6
Risk ratio	0.45	32.88	0.30	112.26	0.40	4.89
Among the "Never-Takers" (35%) [b]						
Risk under no screening[a]	1.1		0.5		13.1	
Risk under screening	0.0	100.0	0.0	100.0	0.0	100.0
Risk difference	-1.1	98.9	-0.5	99.5	-13.1	86.9
Risk ratio	0.00	87.80	0.00	198.81	0.00	7.63
Among the "Compliers" (65%)[a,b]						
Risk under no screening	1.2		0.2		5.6	
Risk under screening	0.8		0.2		5.2	
Risk difference	-0.4		-0.1		-0.4	
Risk ratio	0.69		0.66		0.94	
**Instrumental Conditions and Additive Effect Homogeneity**[a]						
Risk under no screening	1.2		0.3		8.4	
Risk under screening	0.8		0.3		8.0	
Risk difference	-0.4		-0.1		-0.4	
Risk ratio	0.69		0.76		0.96	
**Instrumental Conditions and Multiplicative Effect Homogeneity**[a]						
Risk under no screening	1.2		0.3		8.4	
Risk under screening	0.8		0.2		7.8	
Risk difference	-0.3		-0.1		-0.5	
Risk ratio	0.69		0.66		0.94	

a. Point identification is achieved under these conditions in the NORCCAP trial.

b. In this particular study the distribution of compliance types is known given instrumental conditions. In other study designs, identifying the counterfactual risks and treatment effects within compliance types requires an additional assumption of an assumed feasible distribution of compliance types.

Supplemental Table 4. Age-standardized lower and upper bounds for 10-year counterfactual risks stratified by sex (units= cases/100 persons)

<table>
<tr><th></th><th colspan="2">CRC Incidence</th><th colspan="2">CRC Mortality</th><th colspan="2">All-Cause Mortality</th></tr>
<tr><th></th><th>Lower Bound</th><th>Upper Bound</th><th>Lower Bound</th><th>Upper Bound</th><th>Lower Bound</th><th>Upper Bound</th></tr>
<tr><td>Men (N=49191)</td><td></td><td></td><td></td><td></td><td></td><td></td></tr>
<tr><td><u>No Assumptions</u></td><td></td><td></td><td></td><td></td><td></td><td></td></tr>
<tr><td>Risk under no screening</td><td>1.0</td><td>13.8</td><td>0.3</td><td>13.1</td><td>9.2</td><td>22.0</td></tr>
<tr><td>Risk under screening</td><td>0.1</td><td>87.3</td><td>0.02</td><td>87.2</td><td>0.9</td><td>88.1</td></tr>
<tr><td><u>Instrumental Conditions</u></td><td></td><td></td><td></td><td></td><td></td><td></td></tr>
<tr><td>Overall</td><td></td><td></td><td></td><td></td><td></td><td></td></tr>
<tr><td>Risk under no screening[a]</td><td colspan="2">1.2</td><td colspan="2">0.4</td><td colspan="2">10.2</td></tr>
<tr><td>Risk under screening</td><td>0.5</td><td>39.8</td><td>0.1</td><td>39.4</td><td>4.0</td><td>43.2</td></tr>
<tr><td>Among the "Never-Takers" (39%)[b]</td><td></td><td></td><td></td><td></td><td></td><td></td></tr>
<tr><td>Risk under no screening[a]</td><td colspan="2">1.0</td><td colspan="2">0.4</td><td colspan="2">15.3</td></tr>
<tr><td>Risk under screening</td><td>0.0</td><td>100.0</td><td>0.0</td><td>100.0</td><td>0.0</td><td>100.0</td></tr>
<tr><td>Among the "Compliers" (61%)[a,b]</td><td></td><td></td><td></td><td></td><td></td><td></td></tr>
<tr><td>Risk under no screening</td><td colspan="2">1.4</td><td colspan="2">0.4</td><td colspan="2">6.9</td></tr>
<tr><td>Risk under screening</td><td colspan="2">0.8</td><td colspan="2">0.2</td><td colspan="2">6.5</td></tr>
<tr><td>Women (N=49601)</td><td></td><td></td><td></td><td></td><td></td><td></td></tr>
<tr><td><u>No Assumptions</u></td><td></td><td></td><td></td><td></td><td></td><td></td></tr>
<tr><td>Risk under no screening</td><td>0.9</td><td>14.4</td><td>0.3</td><td>13.7</td><td>6.0</td><td>19.4</td></tr>
<tr><td>Risk under screening</td><td>0.1</td><td>86.7</td><td>0.02</td><td>86.6</td><td>0.6</td><td>87.2</td></tr>
<tr><td><u>Instrumental Conditions</u></td><td></td><td></td><td></td><td></td><td></td><td></td></tr>
<tr><td>Overall</td><td></td><td></td><td></td><td></td><td></td><td></td></tr>
<tr><td>Risk under no screening[a]</td><td colspan="2">1.1</td><td colspan="2">0.3</td><td colspan="2">6.6</td></tr>
<tr><td>Risk under screening</td><td>0.5</td><td>36.5</td><td>0.1</td><td>36.1</td><td>2.6</td><td>38.6</td></tr>
<tr><td>Among the "Never-Takers" (36%)[b]</td><td></td><td></td><td></td><td></td><td></td><td></td></tr>
<tr><td>Risk under no screening[a]</td><td colspan="2">1.3</td><td colspan="2">0.7</td><td colspan="2">10.7</td></tr>
<tr><td>Risk under screening</td><td>0.0</td><td>100.0</td><td>0.0</td><td>100.0</td><td>0.0</td><td>100.0</td></tr>
<tr><td>Among the "Compliers" (64%)[a,b]</td><td></td><td></td><td></td><td></td><td></td><td></td></tr>
<tr><td>Risk under no screening</td><td colspan="2">1.0</td><td colspan="2">0.1</td><td colspan="2">4.4</td></tr>
<tr><td>Risk under screening</td><td colspan="2">0.8</td><td colspan="2">0.2</td><td colspan="2">4.1</td></tr>
</table>

a. Point identification is achieved under these conditions in the NORCCAP trial.

b. In this particular study the distribution of compliance types is known given instrumental conditions. In other study designs, identifying the counterfactual risks and treatment effects within compliance types requires an additional assumption of an assumed feasible distribution of compliance types.

Supplement: Expressions for the counterfactual risks under each level of treatment.

Below we provide bounds for each counterfactual risk, $\Pr[Y^{x=1} = 1]$ (i.e., the risk under treatment) and $\Pr[Y^{x=0} = 1]$ (i.e., the risk under no treatment) where superscripts are used to indicate counterfactuals. In the main text, we presented bounds for the risk difference, i.e., $\Pr[Y^{x=1} = 1] - \Pr[Y^{x=0} = 1]$. Bounds for the risk ratio, $\Pr[Y^{x=1} = 1] / \Pr[Y^{x=0} = 1]$, can be computed by taking the ratio of the lower and upper, and then the upper and lower, bounds of the counterfactual risks below.

Under no assumptions:

$$\Pr[Y = 1|X = 0] \Pr[X = 0] \leq \Pr[Y^{x=0} = 1] \leq \Pr[Y = 1|X = 0] \Pr[X = 0] + \Pr[X = 1]$$

$$\Pr[Y = 1|X = 1] \Pr[X = 1] \leq \Pr[Y^{x=1} = 1] \leq \Pr[Y = 1|X = 1] \Pr[X = 1] + \Pr[X = 0]$$

Under the instrumental conditions:

$$\max\begin{pmatrix} \Pr[Y = 1, X = 0|Z = 0] + \Pr[Y = 1, X = 1|Z = 0] - \Pr[Y = 0, X = 0|Z = 1] - \Pr[Y = 1, X = 1|Z = 1] \\ \Pr[Y = 1, X = 0 \mid Z = 1] \\ \Pr[Y = 1, X = 0 \mid Z = 0] \\ \Pr[Y = 0, X = 1|Z = 0] + \Pr[Y = 1, X = 0|Z = 0] - \Pr[Y = 0, X = 0|Z = 1] - \Pr[Y = 0, X = 1 \mid Z = 1] \end{pmatrix} \leq \Pr\left[Y^{x=0} = 1\right]$$

$$\leq \min\begin{pmatrix} \Pr[Y = 0, X = 1|Z = 0] + \Pr[Y = 1, X = 0|Z = 0] + \Pr[Y = 1, X = 0|Z = 1] + \Pr[Y = 1, X = 1|Z = 1] \\ 1 - \Pr[Y = 0, X = 0 \mid Z = 1] \\ 1 - \Pr[Y = 0, X = 0 \mid Z = 0] \\ \Pr[Y = 1, X = 0|Z = 0] + \Pr[Y = 1, X = 1|Z = 0] + \Pr[Y = 0, X = 1|Z = 1] - \Pr[Y = 1, X = 0 \mid Z = 1] \end{pmatrix}$$

$$\max\begin{pmatrix} \Pr[Y = 1, X = 1|Z = 0] \\ \Pr[Y = 1, X = 1 \mid Z = 1] \\ -\Pr[Y = 0, X = 0|Z = 0] - \Pr[Y = 0, X = 1|Z = 0] + \Pr[Y = 0, X = 0|Z = 1] + \Pr[Y = 1, X = 1|Z = 1] \\ -\Pr[Y = 0, X = 1|Z = 0] - \Pr[Y = 1, X = 0|Z = 0] + \Pr[Y = 1, X = 0|Z = 1] + \Pr[Y = 1, X = 1|Z = 1] \end{pmatrix} \leq \Pr\left[Y^{x=1} = 1\right]$$

$$\leq \min\begin{pmatrix} 1 - \Pr[Y = 0, X = 1|Z = 0] \\ 1 - \Pr[Y = 0, X = 1 \mid Z = 1] \\ \Pr[Y = 0, X = 0|Z = 0] + \Pr[Y = 1, X = 1|Z = 0] + \Pr[Y = 1, X = 0|Z = 1] + \Pr[Y = 1, X = 1|Z = 1] \\ \Pr[Y = 1, X = 0|Z = 0] + \Pr[Y = 1, X = 1|Z = 0] + \Pr[Y = 0, X = 0|Z = 1] + \Pr[Y = 1, X = 1|Z = 1] \end{pmatrix}$$

In the special case when we (i) know there are only "compliers" and "never-takers" and (ii) have no evidence against the exclusion restriction, then within compliance types:

$$\Pr[Y^{x=0} = 1 | X^{z=0} < X^{z=1}]$$

$$= \frac{\Pr[Y = 1 | Z = 0, X = 0] - \Pr[Y = 1 | Z = 1, X = 0] \Pr[X = 0 | Z = 1]}{\Pr[X = 1 | Z = 1]}$$

$$\Pr[Y^{x=1} = 1 | X^{z=0} < X^{z=1}] = \Pr[Y = 1 | Z = 1, X = 1]$$

$$\Pr[Y^{x=0} = 1 | X^{z=0} = X^{z=1} = 0] = \Pr[Y = 1 | Z = 1, X = 0]$$

$$0 \leq \Pr[Y^{x=1} = 1 | X^{z=0} = X^{z=1} = 0] \leq 1$$
